# Factors predictive of clinical outcome in advanced hepatocellular carcinoma patients receiving ramucirumab treatment: A real‐world experience

**DOI:** 10.1002/cam4.6124

**Published:** 2023-06-06

**Authors:** Po‐Ting Lin, Min‐Hua Hung, Shih‐Chieh Shao, Hui‐Yu Chen, Yuk‐Ying Chan, Kai‐Cheng Chang, Shi‐Ming Lin, Huang‐Tz Ou

**Affiliations:** ^1^ Department of Gastroenterology and Hepatology Linkou Chang Gung Memorial Hospital Taoyuan Taiwan; ^2^ College of Medicine Chang Gung University Taoyuan Taiwan; ^3^ Graduate Institute of Clinical Medical Sciences, College of Medicine Chang Gung University; ^4^ Department of Pharmacy Linkou Chang Gung Memorial Hospital Taoyuan Taiwan; ^5^ Department of Pharmacy Keelung Chang Gung Memorial Hospital Keelung Taiwan; ^6^ School of Pharmacy, Institute of Clinical Pharmacy and Pharmaceutical Science, College of Medicine National Cheng Kung University Tainan Taiwan; ^7^ Department of Pharmaceutical Materials Management Chang Gung Medical Foundation Taoyuan Taiwan; ^8^ Department of Pharmacy National Cheng Kung University Hospital Tainan Taiwan; ^9^ School of Pharmacy, College of Medicine National Cheng Kung University Tainan Taiwan

**Keywords:** advanced hepatocellular carcinoma, ramucirumab, real‐world evidence

## Abstract

**Purpose:**

The aim of this study was to investigate the factors predictive of clinical outcome in advanced hepatocellular carcinoma patients receiving ramucirumab treatment.

**Methods:**

We conducted a retrospective study using a multi‐institutional electronic medical records database in Taiwan. We included advanced HCC patients newly receiving ramucirumab as second‐line or beyond systemic therapy between January 2016 and February 2022. The clinical outcomes were median progression‐free survival (PFS) based on the modified Response Evaluation Criteria in Solid Tumors (mRECIST), overall survival (OS) and adverse events. We applied Kaplan–Meier methods to estimate median PFS and OS. Uni‐variable and multi‐variable Cox regression models were applied to identify the prognostic factors.

**Results:**

We included 39 ramucirumab naive users with a median age of 65.5 (IQR: 57.0–71.0) years and treatment time of 5.0 (3.0–7.0) cycles, of whom 82.1% were male and 84.6% were Barcelona Clinic Liver Cancer (BCLC) stage C. After median follow‐up time of 6.0 months, 33.3% of patients' AFP level had decreased more than 20% within 12 weeks. The median PFS and OS were 4.1 months and non‐reach, respectively. Moreover, tumor burden beyond the up‐to‐11 criteria (HR: 2.95, 95% CI: 1.04–8.38) and a decrease in estimated glomerular filtration rate of more than 10% within 12 weeks (HR: 0.31, 0.11–0.88) were significantly related to PFS in the multi‐variable analysis. No patient discontinued ramucirumab during the treatment on account of side effects.

**Conclusion:**

Ramucirumab was an effective treatment option with good AFP response for advanced HCC patients in real‐world experience. Tumor burden beyond the up‐to‐11 criteria and a decrease in estimated glomerular filtration rate were independent predictive factors for progression‐free survival.

## 1 BACKGROUND

Despite improvement in the treatment modalities for hepatocellular carcinoma (HCC), it remains the fourth most common cause of cancer‐related death and the sixth most common incident cases.^1^ The Barcelona Clinic Liver Cancer (BCLC) staging system was developed and has long been used to stratify patients and to guide the best available therapy.^2^ In patients with BCLC stage C, systemic treatment is recommended, with ramucirumab being to date the only systemic treatment guided by a serum biomarker. In subgroup analysis of the REACH trial,^3^ patients with higher AFP levels showed better survival outcomes, although the trial did not reach statistical significance. The REACH‐2 trial^4^ enrolled patients with AFP levels greater than 400 ng/mL and showed significant survival benefits. Ramucirumab was subsequently approved as standard care for second‐line treatment of advanced HCC.

Ramucirumab is a monoclonal antibody with high affinity and specificity targeting vascular endothelial growth factor receptors (VEGFR), an important pathway to inhibit tumor growth which is also widely used in other systemic treatments.^5^, ^6^, ^7^, ^8^, ^9^, ^10^ Purely targeting VEGFR may have less side effects than using tyrosine kinase inhibitor (TKI) and may subsequently prolong the treatment duration. In clinical trials, it was only used as second‐line treatment after sorafenib. However, ramucirumab might be used beyond second‐line treatment, even after other systemic treatments in real‐world practice. Therefore, we aimed to explore the treatment efficacy and safety profile of advanced HCC cases receiving ramucirumab in our hospital to explore the predictors for disease progression and survival.

## 2 METHODS AND MATERIALS

### 2.1 Database

The present study was a retrospective cohort study using the Chang Gung Research Database (CGRD). The CGRD comprises seven hospitals and covers 10% of in‐hospital care and 14% of cancer patients in Taiwan.^11^ The CGRD contains structured and unstructured electronic medical records of all outpatient and inpatient visits, for cancer research. The structured data includes diagnosis, surgery, medication, and laboratory records. The unstructured data includes biopsy, echo, computed tomography (CT), and magnetic resonance imaging (MRI) information. Moreover, the CGRD can be linked to the Taiwan National Cause of Death database and Taiwan Cancer Registry database to mitigate the impact of missing deaths on overall survival (OS) analysis. The CGRD has been proven a reliable database to provide real‐world evidence on cancer research in previous studies.^12^, ^13^ The Institutional Review Board of Chang Gung Medical Foundation (202200830B0) approved this study.

### 2.2 Patients and ramucirumab treatment

We recruited all patients with BCLC stage B or C HCC, newly receiving ramucirumab between January 1, 2016, and February 28, 2022. The first date of ramucirumab use was defined as the index date. Included patients were followed from the index date until loss to follow‐up, death, or May 31, 2022, whichever came first. To avoid short‐term use, we excluded patients who received only one dose of ramucirumab. To evaluate real‐world effectiveness of ramucirumab as a second‐line or beyond treatment, we also excluded patients without baseline imaging records or those combining with other chemotherapy (i.e., cisplatin, fluorouracil and capecitabine).

### 2.3 Outcome assessment

Ramucirumab was administered every 2 weeks with a dosage of 8 mg/kg. All patients received intravenous agents in hospital where nurses recorded any adverse events (AEs) and physicians arranged renal function, liver function, and tumor marker tests at least every 4 weeks, in order to assess the grade of AEs, based on the National Cancer Institute Common Terminology Criteria for Adverse Events (NCI‐CTCAE) version 5.0.^14^ Following National Health Insurance (NHI) rules, patients were required to regularly undergo follow‐up every 2–4 months for tumor response assessment. In our clinical practice, a radiologist assessed liver cancer patients' tumor responses using the modified Response Evaluation Criteria in Solid Tumors (mRECIST) guideline.^15^ In the present study, P‐T Lin and S‐M Lin independently reviewed the imaging database.

### 2.4 Baseline characteristics and prognostic factors

Baseline information regarding prognostic factors including sex, age, weight, Eastern Cooperative Oncology Group (ECOG) performance status, BCLC stage, tumor size, tumor numbers, up‐to‐7 or 11 criteria, presence of macrovascular invasion (MVI) and/or extrahepatic spread (EHS), presence of ascites, Child–Pugh class, albumin‐bilirubin (ALBI) grade and common biochemical examination were recorded at the index date or within the 3 months closest to the index date. Patient HCC etiology, comorbidities, and co‐medication including HBV, HCV, alcohol consumption, Charlson comorbidity index (CCI), and chronic diseases were recorded from 1 year prior to the index date.

### 2.5 Statistical analysis

We described patients' characteristics and analyzed tumor responses and AEs using median and interquartile range (IQR) for continuous variables, and absolute and relative frequencies for categorical variables. We applied the Kaplan–Meier method to estimate median progression‐free survival (PFS) and OS among ramucirumab users based on different prognostic factors. We also applied univariable and multivariable Cox proportional hazard models to estimate hazard ratios (HR) for the different prognostic factors. In the multivariable model, significant variables in the univariable analyses were selected using stepwise regression analysis. We performed all analyses using SAS Enterprise Guide, version 8.3 (SAS Institute).

## 3 RESULTS

### 3.1 Patient characteristics

Within the study period, 94 patients newly commenced ramucirumab treatment. After excluding short‐term uses and chemotherapy combinations, 39 patients remained for further assessment of effectiveness and safety. The details of study population selection are shown in Figure S1. Of these patients, 32 (82.1%) were male with a median age of 65.0 years, and 34 (87.1%) had ECOG performance status less than 2. Among the tumor burdens, 22 (56.4%) and 15 (38.5) met up‐to‐7 and up‐to‐11 criteria, respectively. Table 1 presents a summary of baseline demographic and tumor characteristics.

**TABLE 1:** Baseline characteristics.

	Total (*n* = 39)
Male sex (%)	32 (82.1%)
Age, median years (range)	65.0 (57.0–71.0)
Weight, median kg (range)	65.0 (59.0–71.1)
BMI, median kg/m^2^ (range)	24.4 (22.2–25.8)
ECOG (%)	
0	28 (71.7%)
1	6 (15.4%)
2	5 (12.9%)
Etiology (%)	
HBV	19 (48.7%)
HCV	12 (30.7%)
Alcohol	14 (35.9%)
BCLC stage B/C (%)	6/33 (15.4%/84.6%)
Tumor size (max, cm)	6.8 (3.3–9.1)
Tumor numbers, median (range)	4 (3–7)
Out of up‐to‐7 criteria (%)	22 (56.4%)
Out of up‐to‐11 criteria (%)	15 (38.5%)
MVI (%)	16 (41.0%)
EHS (%)	23 (59.0%)
Ascites (%)	9 (23.1%)
Child–Pugh class A/B (%)	33/6 (84.6%/15.4%)
ALBI grade 1/2/3 (%)	16/20/1 (43.2%/54.1%/2.7%)
Line of treatment (%)	
Second	19 (48.7%)
Third or more	20 (51.3%)
Previous loco‐regional treatment (%)	6 (15.4%)
Within treatment loco treatment (%)	2 (5.1%)
Combined with other systemic treatment (%)	1 (2.6%)
Treatment cycle, median (range)	5.0 (3.0–7.0)
Comorbidity	
Hypertension (%)	14 (35.9%)
Diabetes (%)	8 (20.5%)
Dyslipidemia (%)	4 (10.3%)
Ischemic heart disease (%)	2 (5.1%)
Heart failure (%)	1 (2.6%)
Cerebrovascular disease (%)	1 (2.6%)
COPD (%)	2 (5.1%)
Chronic kidney disease (%)	2 (5.1%)
CCI, mean (range)	4.6 (3–6)
Biochemical data	
AFP (ng/mL)	2662 (750–22829)
Creatinine (mg/dL)	0.84 (0.66–0.98)
eGFR (mL/min/1.73 m^2^)	83.0 (68.0–115.0)
ALT (U/L)	48 (33–71)
AST (U/L)	55 (37–74)
Albumin (g/dL)	3.8 (3.4–4.1)
Total bilirubin (mg/dL)	0.9 (0.7–1.2)
Platelets (10^3^/μL)	122 (87–183)
White blood cell count (10^3^/μL)	5.2 (4.1–7.6)

Abbreviations: ALBI, albumin‐bilirubin; ALT, alanine aminotransferase; AST, aspartate aminotransferase; BCLC, Barcelona Clinic Liver Cancer; BMI, body mass index; CCI, Charlson comorbidity index; COPD, chronic obstructive pulmonary disease; ECOG, Eastern Cooperative Oncology Group; eGFR, estimated glomerular filtration rate; EHS, extrahepatic spread; MVI, macrovascular invasion.

*Note*: Continuous variables are expressed as median (interquartile range) and dichotomous variables are expressed as percentage (%).

### 3.2 Real‐world efficacy and safety of ramucirumab

**FIGURE 1:**
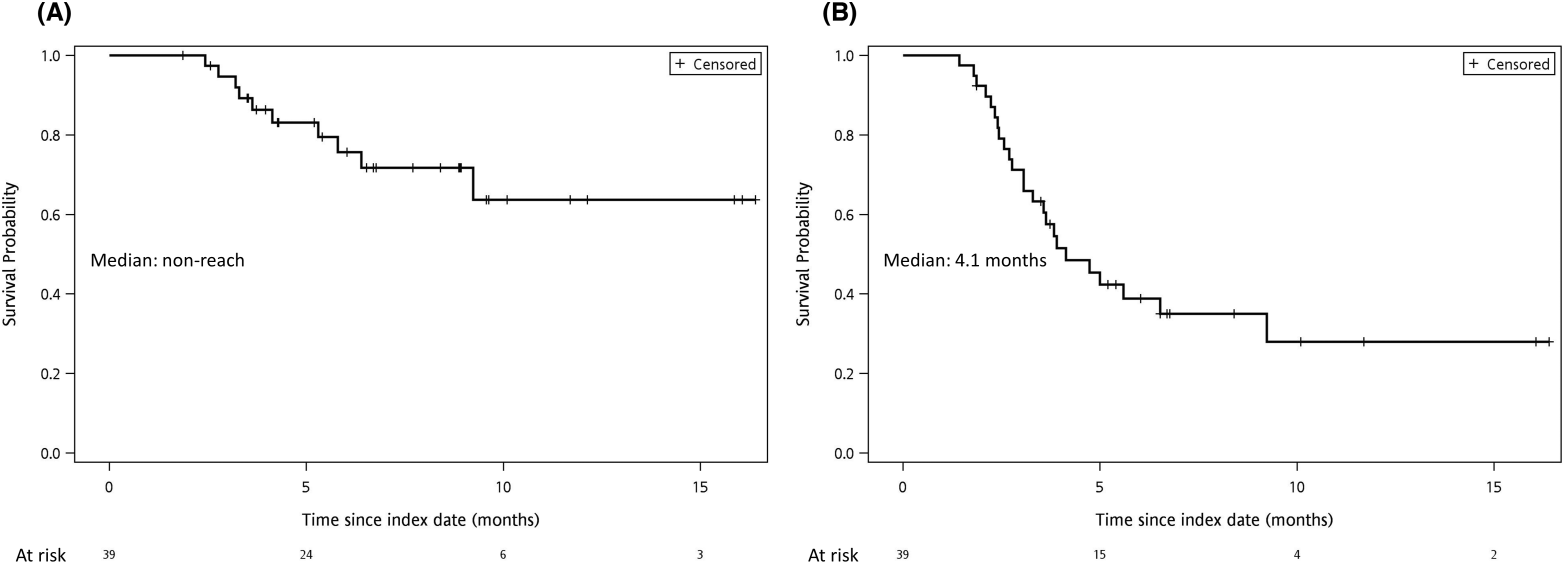
Kaplan–Meier curve of overall survival and progression‐free survival among advanced HCC patients under ramucirumab treatment: (A) overall survival; (B) progression‐free survival.

**FIGURE 2:**
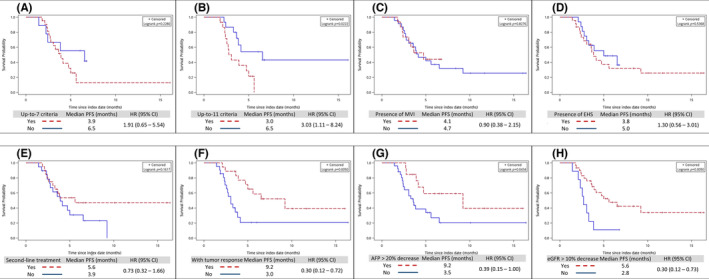
Kaplan–Meier curve of progression‐free survival between different subgroup: (A) by up‐to‐7 criteria; (B) by up‐to‐11 criteria; (C) by presence of macrovascular invasion; (D) by presence of extrahepatic spread; (E) by second or more line of treatment; (F) by tumor response (with partial response or stable disease); (G) by AFP decrease more than 20%; (H) by eGFR decrease more than 10%.

During the median 6.0 months follow‐up, 3 (7.7%) and 15 (38.5%) patients had partial response (PR) and stable disease (SD), respectively. Among all patients, the disease progression rate was 41.0%. Of the 39 patients, 13 (33.3%) showed a decline in AFP of more than 20%. Furthermore, the median PFS and OS were 4.1 months and non‐reach, respectively (Figure 1). Figure 2 summarizes the different subgroup analyses of median PFS, whereby the HCC patients with up‐to‐11 criteria had worse PFS (3.0 vs. 6.5 months, *p* < 0.05). Otherwise, patients with AFP decreased by more than 20% had better PFS (9.2 vs. 3.5 months, *p* < 0.05), and those with eGFR decreased by more than 10% also had better PFS (5.6 vs. 2.8 months, *p* < 0.01).

**FIGURE 3:**
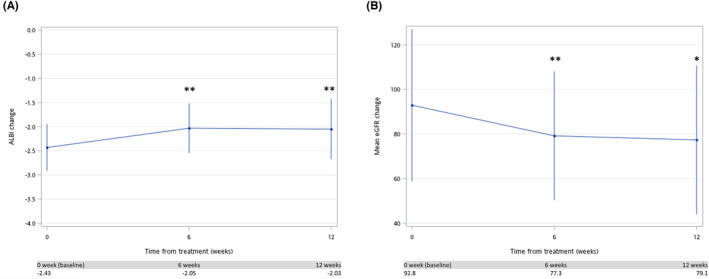
AFP and eGFR change from baseline every 6 weeks: (A) AFP; (B) eGFR. Index date refers to the first date of initiation of ramucirumab. *p < 0.05; **p < 0.01; ***p < 0.001.

As regards the safety evaluation, treatment‐related ascites and eGFR decrease occurred in 20 (51.3%) and 31 (79.5%) patients, respectively. AEs for any reason were reported in 37 patients (94.9%), whereby the most common AE was edema (14, 35.9%). These AEs were of grade 2 in four patients (10.2%) and none of the patients reported events of grade 3–5. Table 2 presents the details of tumor responses and AEs among patients receiving ramucirumab treatment. Moreover, Figure 3 shows the changes in ALBI score and eGFR every 6 weeks. Compared to baseline, the ALBI score significantly increased after ramucirumab treatment (−2.43 vs. −2.05, *p* < 0.001). As regards eGFR dynamic change, the eGFR significantly decreased after ramucirumab treatment (92.8 vs. 79.1, *p* < 0.05).

**TABLE 2:** Tumor response and adverse events after ramucirumab treatment.

	Number (%)
Tumor response	
Partial response	3 (7.7)
Stable disease	15 (38.5)
Objective response rate	3 (7.7)
Disease control rate	18 (46.2)
Disease progression	16 (41.0)
AFP decline 20%	13 (33.3)
Adverse events	
Post treatment ascites	20 (51.3)
eGFR decline 10%	31 (79.5)
Edema	14 (35.9)
Skin rash	7 (17.9)
Constipation	1 (2.5)
Vomiting	5 (12.8)
Diarrhea	4 (10.2)
Fatigue	9 (23.0)
Hand foot syndrome	2 (5.1)
Fullness	3 (7.6)
Hypertension	1 (2.5)
Anorexia	4 (10.2)
Fever	5 (12.8)
Weak	4 (10.2)
Abdominal pain	4 (10.2)
Headache	3 (7.6)
Poor appetite	3 (7.6)
Others	22 (56.4)
Any grade 1	37 (94.9)
Any grade 2	4 (10.2)

Abbreviation: eGFR, estimated glomerular filtration rate.

### 3.3 Prognostic factor analyses for PFS

In the univariable Cox regression analysis, we found that out of the up‐to‐11 criteria, better tumor response, decrease of AFP and eGFR were significantly associated with better PFS. For the multivariable analysis, we excluded the variable of tumor response in the model due to the surrogate endpoint of PFS. Finally, up‐to‐11 criteria (HR: 2.95, 95% confidence interval [CI]: 1.04–8.38) and eGFR decrease of 10% (HR: 0.31, 95% CI: 0.11–0.88) were identified as independent prognostic factors for PFS (Table 3).

**TABLE 3:** Univariable and multivariable analysis of potential predictive factors for progression‐free survival.

	Univariable			Multivariable		
Variable	HR	95% CI		HR	95% CI	
Age ≥65	0.87	0.38	1.98			
Sex (male)	0.52	0.17	1.56			
ECOG ≥2	1.87	0.54	6.40			
BCLC stage B	1.31	0.48	3.53			
Out of up‐to‐7	1.91	0.65	5.54			
Out of up‐to‐11	3.03	1.11	8.24	3.42	1.22	9.58
Presence of MVI	0.90	0.38	2.15			
Presence of EHS	1.30	0.56	3.01			
ALBI grade 2	1.24	0.53	2.89			
Child–Pugh A	0.73	0.24	2.19			
HBV	2.22	0.96	5.10			
HCV	0.94	0.37	2.39			
Alcohol	0.57	0.23	1.38			
Post ascites	1.13	0.50	2.57			
As second treatment	0.73	0.32	1.66			
Previous TKI	0.48	0.14	1.66			
With previous sorafenib	0.57	0.23	1.39			
Previous ICIs	1.26	0.56	2.81			
Tumor response: CR + PR	0.37	0.05	2.75			
Tumor response: CR + PR + SD	0.30	0.12	0.72			
AFP >20% decrease	0.39	0.15	1.00			
eGFR >10% decrease	0.30	0.12	0.73	0.32	0.12	0.89

Abbreviations: ALBI: albumin‐bilirubin; BCLC: Barcelona Clinic Liver Cancer; CCI: Charlson comorbidity index; CR: complete response; ECOG: Eastern Cooperative Oncology Group; eGFR: estimated glomerular filtration rate; EHS: extrahepatic spread; ICI: immune checkpoint inhibitor; MVI: macrovascular invasion; PR: partial response; SD: stable disease; TKI: tyrosine kinase inhibitor.

## 4 DISCUSSION

The current retrospective study showed that ramucirumab, a monoclonal antibody targeting the VEGFR receptor‐2 was an effective treatment option for advanced HCC patients in real‐world experience. Despite the tolerable safety profile, liver function fluctuation during treatment should be monitored.

Vascular endothelial growth factor (VEGF) is one of the critical molecular targets in cancer treatment and has been used in several successful pivotal combination regimens for not only HCC but also other cancers.^16^, ^17^, ^18^, ^19^, ^20^, ^21^, ^22^, ^23^, ^24^ The tumor stays alive by secretion of VEGF to promote angiogenesis for oxygen and nutrient supply. Therefore, blocking VEGF can theoretically prevent tumor growth, tissue invasion, and metastasis. Since 2008, there have been multiple systemic treatment choices for HCC, including sorafenib,^5^ lenvatinib,^6^ atezolizumab plus bevacizumab,^10^ regorafenib,^7^ cabozantinib,^9^ and ramucirumab.^3^ All the regimens above block the VEGF pathway as one target, in turn highlighting the importance of this pathway. In previous reports, anti‐VEGF agents were able to modulate immune function and enhance T cell antitumor activity,^25^, ^26^ which might explain why single agents with an immune checkpoint inhibitor (ICI) such as pembrolizumab and nivolumab failed to reach statistical significance,^27^, ^28^ while combining an anti‐VEGF agent with ICI improved clinical outcomes significantly.^10^ This points to the immunomodulator effect of the anti‐VEGF agents. Therefore, ramucirumab followed by ICI might have a greater treatment impact than if followed by TKI. A Japanese group has also pointed out this phenomenon of outstanding treatment response when followed by atezolizumab plus bevacizumab.^29^ However, in our study, there was no significant difference in outcome when ramucirumab was given after ICI. One of the reasons was that the patients were all treated with TKI before ICI. Larger, prospective studies in the future are warranted to confirm and compare effective treatment algorithms with ramucirumab followed by ICI or TKI.

Higher AFP level correlated with higher expression of the VEGFR2 receptor.^30^ In theory, ramucirumab as a very potent anti‐VEGFR2 medication is more beneficial for HCC patients with higher AFP levels, which explains the success of the REACH‐2 study. Although a barely satisfactory objective response rate was expected when used as a single agent for second‐line treatment, ramucirumab showed good disease control rate and on‐treatment AFP response in the REACH and REACH‐2 cohorts.^4^, ^31^ In real‐world experience,^29^, ^32^, ^33^ ramucirumab has been used after systemic treatment other than sorafenib and has been used for more than second‐line treatment. Kuzuya et al. found that AFP level decreased at 2 weeks in 50% of patients and the disease control rate was 80%, in those receiving ramucirumab after lenvatinib.^32^ Later, the same group also confirmed ramucirumab as an effective treatment option after atezolizumab plus bevacizumab, with an objective response rate up to 15% and a decrease in AFP level in up to 84.6% of patients within 2 weeks.^29^ Likewise, up to one third of patients in our cohort had a decrease in AFP level of more than 20%. Therefore, the current study confirmed the treatment efficacy of ramucirumab for second‐line and beyond treatment with a large sample size in Taiwan.

Until now, ramucirumab has been the only systemic treatment with biomarker “AFP”. AFP level correlates with tumor burden in HCC patients.^34^ On account of the inclusion criteria of the trial, the ramucirumab cohort was prone to enrolling patients with larger tumor burden. Therefore, “up‐to‐seven” criteria^35^, ^36^ could not fit this group of patients well. In our cohort, patients beyond “up‐to‐11 criteria” tended to have poorer PFS and this was then one of the independent risk factors for PFS (HR 2.95). Due to the high possibility of tumor progression in patients beyond up‐to‐11 criteria, clinicians should carefully monitor tumor condition. Early shifting to sequential treatment or using a combination regimen should be considered for patients with high tumor burden beyond up‐to‐11 criteria.

Owing to the simplicity of targeting the receptors, ramucirumab has a good side effect profile. In the REACH‐2 trial, the incidence of AEs was low with good maintenance of liver function during treatment.^4^ Maintaining liver function was the key to better survival. One study showed no deterioration of liver function during ramucirumab treatment after lenvatinib, but another study revealed worse liver function during ramucirumab treatment after atezolizumab plus bevacizumab.^29^, ^32^ In our cohort, the ALBI score worsened during treatment. The probable reason was that we enrolled patients receiving ramucirumab beyond second‐line treatment, as in the study.^29^ The baseline liver function might have been worse if patients had received multiple systemic treatments. This means, even though the safety profile has been validated in the trial, we must still be attentive to the patient's liver function and try our best to maintain it. Of note, the anti‐VEGF agent was shown to alter glomerular filtration in previous literature,^37^, ^38^, ^39^ and ramucirumab was also shown to have an impact on renal function in some case studies.^40^, ^41^ In our cohort, a significant decrease in estimated glomerular filtration rate was noted during ramucirumab treatment. Although no patient discontinued treatment or required hemodialysis under treatment, monitoring renal function was essential. An association between side effects and treatment response has been reported previously in some systemic treatments.^42^, ^43^, ^44^, ^45^ In our study, a decrease in glomerular filtration rate was one of the independent predictive factors for PFS. Because a decrease in renal function is one side effect of anti‐VEGF drugs, patients who experience this side effect might have better treatment response under ramucirumab than those who receive other systemic treatment.^42^, ^43^, ^44^, ^45^ However, further study is required to verify our results and we should pay attention to the patient's renal function during treatment.

There were some limitations to the current study. First, it was a retrospective and non‐randomized study. Selection bias was inevitable. Second, the cohort size was small and included patients with systemic treatment beyond second line. Third, follow‐up duration was not long enough to evaluate the OS.

In conclusion, ramucirumab was an effective treatment option with good AFP response for advanced HCC patients in real‐world experience. Tumor burden beyond up‐to‐11 criteria and the decrease in estimated glomerular filtration rate were independent predictive factors for progression‐free survival. Despite the safety profile being tolerable, liver function fluctuations and renal function during treatment require ongoing monitoring.

## AUTHOR CONTRIBUTIONS

**Po‐Ting Lin:** Conceptualization (equal); data curation (equal); writing – original draft (equal); writing – review and editing (equal). **Min‐Hua Hung:** Data curation (equal). **Shih‐Chieh Shao:** Conceptualization (equal); formal analysis (equal); methodology (equal); writing – original draft (equal); writing – review and editing (equal). **Hui‐Yu Chen:** Project administration (equal); resources (equal). **Yuk‐Ying Chan:** Project administration (equal); resources (equal). **Kai‐Cheng Chang:** Conceptualization (equal); data curation (equal); formal analysis (equal); methodology (equal); software (equal); writing – original draft (equal); writing – review and editing (equal). **Shi‐Ming Lin:** Methodology (equal); project administration (equal); writing – review and editing (equal). **Huang‐Tz Ou:** Supervision (equal); writing – review and editing (equal).

## FUNDING INFORMATION

This study was supported by grants from Chang Gung Medical Research Fund (CMRPG3M039).

## CONFLICT OF INTEREST STATEMENT

None reported.

## ETHICS STATEMENT

The datasets generated and/or analyzed during the current study are not publicly available for reasons of data privacy.

## CONSENT

Informed consent requirement was waived due to the retrospective study design.

## PERMISSION TO REPRODUCE MATERIAL FROM OTHER SOURCES

Not applicable.

## CLINICAL TRIAL REGISTRATION

Not applicable.

## Supporting information


Figure S1.

## Data Availability

The datasets generated during and/or analyzed during the current study are not publicly available due data privacy.
