# Supplementary figures and images for: Factors predictive of clinical outcome in advanced hepatocellular carcinoma patients receiving ramucirumab treatment: A real‐world experience
Source: Cancer Med. 2023 Jun 6;12(14):14902–11. doi: 10.1002/cam4.6124 (PMC10417170; doi:10.1002/cam4.6124)

**Supplemental Figure 1. Selection of study cohort from database**

**
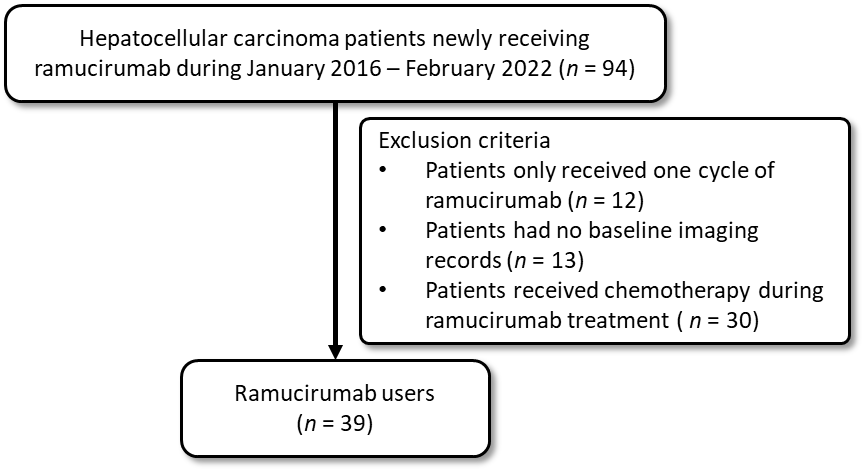
**

Supplement: Supplementary file 1 — Figure S1. [file CAM4-12-14902-s001.docx]
